# Supplementary material for: Chromothripsis during telomere crisis is independent of NHEJ, and consistent with a replicative origin
Source: Genome Res. 2019 May;29(5):737–49. doi: 10.1101/gr.240705.118 (PMC6499312; doi:10.1101/gr.240705.118)
Supplement: Supplemental Material [file supp_gr.240705.118_Supplemental_file_1.zip › contigs/annotated_contigs/DB114/contig.2.DB114_length_529_mean_cov_8.73724007561.docx]

**DB114_length_529_mean_cov_8.73724007561**

```
ATGTTTCTCTTCTATTCCGTGTGAAATTCAGTCTATCCCCAGAGTGGTCACTAGGCATACATTCTCACCTCTTCTCTTTCCACTAACAT
 >chr18:49857579-49857878 + E=2e-165
GACCATAAAAAGCCATTTTCTTAGTTTCTCCACTCAGGCCTACTTCCTCACAGAGTTGAGCTCTAACAACTTGCAATTCTTTCCAAACC

ATTAAATATACATACAGATATGTATGTGTATATATATATATACACGCACACACACACACATATATAATATATATATACACACACAGATA

GATGTATATTTAATGGTTTGGAAAGAGTTGCA|T|AAAATCATAAATTTTAAAGTGGGAAAATGCACTTATTAAATAGAATCCTTGTTT
                                  >chr18:49859935-49860164 + E=4e-123
TTAATTAGTTTAATTAGAATGCTTCATGTTGTAAAATGTATATATTCTCCTTAACCTGGATTAAATAATAAAAGAATTAATTGACCCCT

ATAAGTGAGAAATCCAAAGGCAGGGATGGCTTTAGGTTAGATGGCTTATTCCAATGACCCAAATAATGTAACCAAAATCTCAGTTT
```
